# Prospective Evaluation of *Aspergillus fumigatus*-Specific IgG in Patients With Cystic Fibrosis

**DOI:** 10.3389/fcimb.2020.602836

**Published:** 2021-01-22

**Authors:** Patience Eschenhagen, Claudia Grehn, Carsten Schwarz

**Affiliations:** Department of Pediatric Pneumology, Immunology and Intensive Care Medicine, CF Center, Charité—Universitätsmedizin Berlin, Berlin, Germany

**Keywords:** cystic fibrosis, aspergillosis, *Aspergillus fumigatus*, allergic bronchopulmonary aspergillosis, ABPA, Gm3, *Aspergillus fumigatus*-specific IgG

## Abstract

**Background:**

In Cystic Fibrosis (CF), the airways are often colonized by opportunistic fungi. The most frequently detected mold is *Aspergillus fumigatus* (*Af*). *Af* diseases are associated with significant morbidity and mortality. The most common clinical picture caused by *Af* is allergic bronchopulmonary aspergillosis (ABPA), triggered by an immunological reaction against *Af*. *Af* bronchitis and invasive aspergillosis rarely occur in CF as a result of spore colonization and germination. Since pulmonary mycoses and exacerbations by other pathogens overlap in clinical, radiological, and immunological characteristics, diagnosis still remains a challenge. The search for reliable, widely available biomarkers for *Af* diseases is therefore still an important task today.

**Objectives:**

*Af-*specific IgG m3 is broadly available. Sensitivity and specificity data are contradictory and differ depending on the study population. In our prospective study on pulmonary *Af* diseases in CF, we determined specific IgG m3 in order to test its suitability as a biomarker for acute *Af* diseases and as a follow-up parameter.

**Methods:**

In this prospective single center study, 109 patients with CF were screened from 2016 to 2019 for *Af*-associated diseases. According to diagnostic criteria, they were divided into four groups (control, bronchitis, ABPA, pneumonia). The groups were compared with respect to the level of *Af*-specific IgG (ImmunoCAP Gm3). We performed a receiver operating characteristic (ROC) curve analysis to determine cut-off, sensitivity and specificity. Twenty-one patients could be enrolled for a follow-up examination.

**Results:**

Of the 109 patients, 36 were classified as acute *Af*-disease (*Af* bronchitis, ABPA, *Af* pneumonia). Of these, 21 patients completed follow up-screening. The median *Af*-specific Gm3 was higher in the acute *Af*-disease groups. There was a significant difference in *Af*-specific IgG m3 compared to the control group without acute *Af*-disease. Overall, there was a large interindividual distribution of Gm3. A cut-off value of 78.05 mg/L for Gm3 was calculated to discriminate controls and patients with ABPA/pneumonia with a specificity of 75% and a sensitivity of 74.6%. The follow up examination of 21 patients showed a decrease of Gm3 in most patients without statistical significance due to the small number of follow up patients.

**Conclusion:**

*Af *specific IgG may be a useful biomarker for acute ABPA and *Af *pneumonia, but not for *Af* bronchitis in CF. However, due to the large interindividual variability of Gm3, it should only be interpreted alongside other biomarkers. Therefore, due to its broad availability, it could be suitable as a biomarker for ABPA and *Af* pneumonia in CF, if the results can be supported by a larger multicenter cohort.

## Introduction

Cystic fibrosis (CF) is the most common lethal genetic disease in Caucasians and affects approximately between 70,000 and 100,000 people worldwide. The leading cause of death is the progressive lung disease, driven by a complex and diverse inflammatory immune syndrome induced by recurrent and chronic bacterial and fungal infections of the lung (Stephenson et al., 2015; Bell et al., 2020).

*A. fumigatus (Af)*, the most frequently detected mold fungus in CF airways, is the fungal pathogen with the most impact on morbidity and mortality in CF (Pihet et al., 2009; Amin et al., 2010; Middleton et al., 2013; Schwarz et al., 2018a). The defective chloride channel in CF together with impaired mucociliary clearance and structural changes resulting from infectious complications (e.g. cavitary lung disease, bronchiectasis) result in a local mucosal immunodeficiency (Cohen and Prince, 2012), which allows airway pathogens like *Af* conidia to colonize and germinate in the airways. Allergic bronchopulmonary aspergillosis (ABPA), bronchitis, aspergilloma and invasive infection (pneumonia) are the known disease entities caused by *Af* in CF (Moss, 2010; Baxter et al., 2013a; Middleton et al., 2013; Hong et al., 2018).

ABPA is an immune mediated lung disease with a prevalence of up to 15% in CF. It manifests with a poorly controlled obstructive disease and recurrent pulmonary infiltrates with or without bronchiectasis (Stevens, 2003). According to the 2003 CFF (Cystic Fibrosis Foundation) consensus criteria, classic diagnosis of ABPA comprises clinical deterioration not attributed to other causes, serum IgE >1000 IU/ml, a positive skin prick test or positive specific IgE, antibodies against *Af*, precipitating or IgG antibodies against *Af*, and radiological changes that are not treatable by antibiotics. For the minimal ABPA criteria, serum IgE >500 IU/ml and fewer of the criteria mentioned above are sufficient to suspect ABPA (Stevens, 2003). Invasive pulmonary *Af* infections primarily occur in severely immunocompromised patients, but they also can occur in CF if additional risk factors are present (e.g. severe lung disease, diabetes, steroid therapy) (Kousha et al., 2011; Hong et al., 2018). The prevalence is extremely low, thus precise data are not available. In CF, invasive pulmonary *Af* infections often show a subacute, slowly progressing clinical course. and is induced by local invasion of *Af (*Schwarz et al., 2018b). Radiographic imaging and clinical courses are often non-specific (Felton and Simmonds, 2014; Kosmidis and Denning, 2015). This bears the risk of delayed diagnosis in CF. *Af* bronchitis is another *Af* entity with an estimated prevalence of 9% in CF. Increasing evidence exists that *Af* in CF can cause airway symptoms without hypersensitivity and without tissue invasion of fungal hyphae (Felton and Simmonds, 2014; Brandt et al., 2018). Shoseyov et al. were the first to report on *Af* bronchitis in six patients (Shoseyov et al., 2006). The group suggested that bronchitis should be considered, and antifungal therapy initiated when deteriorating respiratory function is not responding to antibacterial therapy, *Af* is growing in sputum cultures and ABPA was excluded. Chrdle et al. proposed a definition using clinical criteria: symptomatic chronic lower airway disease, detection of *Af* in sputum or bronchial alveolar lavage (BAL) by culture or PCR, and detection of Aspergillus-specific IgG antibodies (Chrdle et al., 2012). In summary, repeatedly detected *Af* in sputum samples and persistent respiratory symptoms without signs of *Af* hypersensitivity and without radiographic evidence of infection are characteristics of *Af* bronchitis in patients with CF (Felton and Simmonds, 2014; Kosmidis and Denning, 2015). Due to the polymicrobial colonization, overlapping clinical, radiographic, and laboratory characteristics, it is still difficult to clearly distinguish *Af* conditions from other causes of bronchopulmonary exacerbation in CF (Stevens, 2003). This bears the risk of *Af* diseases being missed and can lead to situations in which a potentially harmful differential therapeutic strategy must be attempted in order to achieve successful treatment. Therefore, the search for reliable, widely available biomarkers for *Af* diseases is still an important task today (el-Dahr et al., 1994; Kurup, 2005; Latzin et al., 2008; Hafen GM et al., 2009; Delhaes et al., 2010; Scheffold et al., 2018; Keown et al., 2019). In this light, it seems useful to evaluate single biomarkers in terms of their significance for *Af* diseases. *Af* IgG antibodies are used for diagnosis of chronic pulmonary aspergillosis (CPA) and aspergillus diseases in patients with chronic lung disorders and are referred to as diagnostic criteria in many guidelines (Stevens, 2003; Delhaes et al., 2010; Agarwal et al., 2017). They are found in many patients with ABPA, bronchitis and invasive aspergillosis, and several assays are available. In the clinical routine, in recent years, technologies like ImmunoCAP have continuously replaced older methods like immunoprecipitation (Van Hoeyveld et al., 2006; Baxter et al., 2013b). The ImmunoCAP technique provides quantitative measures and reproducible results and therefore a higher sensitivity and specificity in detecting aspergillus conditions (Baxter et al., 2013b). However, there is still a lack of standardization in terms of cut-off values, as several working groups examining *Af* IgG in *Af* related conditions obtained discrepant results, which is in part due to different study designs and different laboratory methods used, but this may also reflect the different host-pathogen-interaction of heterogeneous study populations with different underlying diseases (Agarwal et al., 2017; Sehgal et al., 2018; Alghamdi et al., 2019; Sehgal et al., 2019). We therefore evaluated specific *Af* IgG in ABPA, *Af* bronchitis and invasive aspergillosis in CF.

## Materials and Methods

This prospective study was conducted between January 2016 and December 2019 at Christiane Herzog Cystic Fibrosis Center, Charité—Universitätsmedizin Berlin, Germany. Ethical aspects were considered and approval for the study was gained by the Ethics Committee of Charité—Universitätsmedizin Berlin, Germany (Number EA2/121/16).

We collected sera from 109 patients with CF, ages 6–69 years, independent of their history of *Af* related conditions. To obtain both a control group and a disease group, *Af*-specific IgG was determined either as part of a diagnostic workup when *Af* conditions were suspected or without such suspicion in pulmonary exacerbated and in clinically stable patients during routine checkup. *Af*-specific IgG (ImmunoCAP Gm3) was determined in our routine laboratory. Depending on the diagnosis criteria for *Af* related conditions in CF, the patients were categorized into four groups: control, *Af* bronchitis, ABPA, and *Af* pneumonia. As a few patients were assessed several times during the course of the study, some follow-up measurements for Gm3 are available and listed below.

### Diagnosis Criteria for *Af* Related Conditions

Diagnosis of ABPA was based on the minimal 2003 Cystic Fibrosis Foundation (CFF) consensus criteria: i) serum IgE >500 IU/ml; ii) *Af*-specific IgE >0.35 kU/L; iii) clinical deterioration not attributed to other causes; and one of the following criteria: i) presence of *Af* IgG (Gm3 ImmunoCAP) antibodies in serum; ii) new or recent abnormalities on chest radiography or chest CT that have not cleared with antibiotics and standard physiotherapy. In one patient, diagnosis of ABPA was assumed despite a total IgE <500 kU/L due to a threefold increase in total IgE and positive diagnosis criteria.

*Af* bronchitis was defined as: i) clinical deterioration with repeatedly positive sputum and *Af* PCR or repeatedly positive sputum cultures (≥2 during the past 6 months) for *Af*, ii) no antibiotic treatment response, iii) total serum IgE level <200 kU/L, iv) no observation of new pulmonary infiltrates, and v) appropriate antifungal treatment response.

Criteria for diagnosis of *Af* pneumonia were i) positive sputum *Af* PCR or repeatedly positive sputum cultures for *Af*, ii) new or recent abnormalities on chest radiography or chest CT that have not cleared with antibiotics and standard physiotherapy, iii) no antibiotic treatment response, and iv) total serum IgE level <200 kU/L. In two patients, diagnosis of pneumonia was postulated due to repeatedly positive sputum cultures for *Af* combined with typical radiographic features.

Subjects were excluded if they met any of the following criteria: i) intake of systemic glucocorticoids >5 days for ABPA diagnosis and/or antifungal therapy >5 days within the last 4 weeks; ii) failure to provide informed consent. Patients on standard therapy for *Af* diseases in CF (corticosteroid therapy and/or antifungal treatment) were excluded to enable a clear diagnosis and follow up examination after therapy.

For the available follow up measurements of Gm3, data >30 and <180 days after diagnosis and initiation of therapy were included if the patients were stable at that time and did not meet the diagnostic criteria for *Af* disease.

### *Af*-Specific IgG (Gm3)

*Af*-specific IgG (Gm3) concentration was measured in our routine laboratory by the commercial ImmunoCAP system (Phadia, Thermo Fisher Scientific, USA), which uses automated fluorescent enzyme immunoassay (FEIA) technology. The m3 antigen is a crude extract from whole *Af* conidia and mycelia. According to manufacturer data, the reference value is <39 mg/L.

### Statistical Analysis

Median and ranges were calculated for metrical variables. Distribution of data was assessed with Shapiro–Wilk test for normal distribution. For comparison of two groups *t* test or Mann–Whitney test was applied as appropriate. Comparison of more than two groups with normally distributed data sets was performed with one-way ANOVA including Tukey´s multiple comparison test. Not normally distributed data were analyzed by Kruskal Wallis test. Frequency and percentage were used for categorical variables. Associations of categorical variables were analyzed using chi-square test. Cut-off value determination for IgGm3 was based on receiver operating characteristic (ROC) analysis. A *P* value < 0.05 was accepted to indicate statistical significance. Data analysis were performed with GraphPad Prism version 8 (GraphPad Software).

## Results

### Baseline Characteristics

One hundred nine sera of patients with CF were analyzed for *Af* IgG (Gm3 ImmunoCAP), with 63 patients assigned to the control group, 22 patients to the bronchitis group, 18 patients to the ABPA group and six patients to the pneumonia group. The median age of all participants was 26 years (range: 6–69 years). The median BMI was 19.8 kg/m^2^, with the lowest BMI in the pneumonia group (17.5 kg/m^2^). The median percent predicted FEV_1_ was 54 (range: 16.0–133.1), with the highest median percent predicted FEV_1_ in the ABPA group (60) and the lowest median percent predicted FEV_1_ in the pneumonia group (22, p < 0,01). 24.8% of the patients had diabetes, with the highest proportion in the pneumonia group (66.7%, no statistical significance). The mean total IgG was 13.2 kU/L and was markedly higher in the pneumonia group (16.3 kU/L, no statistical significance). Co-colonization with *Candida* spp. was documented in 85.3% of all patients (control group 84.1%, bronchitis 90.9%, ABPA and pneumonia each 83.3%, no statistical significance). Detection of other fungal pathogens was documented in 11% of all patients (22.7% bronchitis, 5.6% ABPA, 0% pneumonia, no statistical significance) (see **Table 1**).

**Table 1:** Clinical characteristics of included patients.

*Variable	Total	Control	Bronchitis	ABPA	Pneumonia	*P*-value
Number of patients, n	109	63	22	18	6	
Age, years, median (range)	26.0 (6–69)	27.0 (6–69)	26.0 (8.0–57.0)	21.5 (9.0–49.0)	30.5 (15.0–43.0)	ns
Female sex, n (%)	62 (56.9%)	37 (58.7%)	14 (63.6%)	9 (50.0%)	2 (33.3%)	ns
CFTR dF508 homozygous, n (%)	49 (45.0%)	26 (41.3%)	12 (54.5%)	8 (44.4%)	3 (50.0)	ns
BMI, kg/m^2^, median (range)	19.8 (12.4–34.2)	20.2 (12.4–34.2)	20.2 (14.5–29.5)	18.8 (14.6–26.9)	17.5 (15.3–23.5)	ns
Percent-predicted FEV1, median (range)	54.0 (16.0–133.1)	60.0 (17.0–133.1)	41.0 (16.3–96.6)	60.0 (27.0–90.0)	22.0 (16.0–57.0)	<0.01
Diabetes, n (%)	27 (24.8%)	12 (19.1%)	6 (27.3%)	5 (27.8%)	4 (66.7%)00	ns
Pancreatic insufficiency, n (%)	100 (91.7%)	57 (90.5%)	20 (90.9%)	17 (94.4%)	6 (100%)	ns
Total IgG, kU/L, median (range)	13.2 (2.0–73.0)	13.2 (6.0–73.0)	12.3 (2.0–21.9)	13.6 (6.2–25.2)	16.3 (13.0–22.0)	ns
Gm3, mg/L, median (range)	60.0 (3.0–200)	48.9 (2.7–200)	51.2 (18.2–145)	97.5 (24.2–200)	95.3 (78.2–140)	<0.01
Gm3 > 39 mg/L, n (%)	72 (66,1)	37 (58,7)	15 (68,2)	14 (77,8)	6 (100)	
Total IgE, kU/L, median (range)	34.5 (2.0–4359)	26.5 (2.5–199)	30.2 (2.1–262)	1048 (344–4359)	44.4 (2.0–84.5)	<0.01
*Aspergillus* specific IgE, kU/L, median (range)	0.1 (0.1–51.7)	0.1 (0.1–0.1)	0.1 (0.1–3.2)	25.7 (10.8–51.7)	0.1 (0.1–1.0)	<0.01
Steroids, n (%)	17 (15.6%)	0 (0%)	8 (36.4%)	7 (38.9%)	2 (33.3%)	<0.01
Antifungal treatment (Caspofungin, Itraconazole, Posaconazole), n (%)	8 (7.3%)	0 (0%)	2 (9.1%)	5 (27.8%)	1 (16.7%)	<0.01
Chronic *Pseudomonas aeruginosa*, n (%)	71 (65.1%)	37 (58.7%)	17 (77.3%)	13 (72.2%)	4 (66.7%)	ns
*Aspergillus fumigatus* positive sputum, n (%)	45 (41.3%)	21 (33.3%)	17 (77.3%)	5 (27.8%)	2 (33.3%)	<0.01
*Aspergillus* PCR positive, n (%), n (%)	60 (55.0%)	30 (47.6%)	16 (72.7%)	12 (66.7%)	2 (33.3%)	ns
*Candida* spp. positive sputum, n (%)	93 (85.3%)	53 (84.1%)	20 (90.9%)	15 (83.3%)	5 (83.3%)	ns
Detection of other fungal pathogens*, n (%)	12 (11.0%)	6 (9.5%)	5 (22.7%)	1 (5.6%)	0 (0%)	ns

*Except Candida and Aspergillus species.

### Af-Specific IgG (Gm3) Results

**Figure 1:**
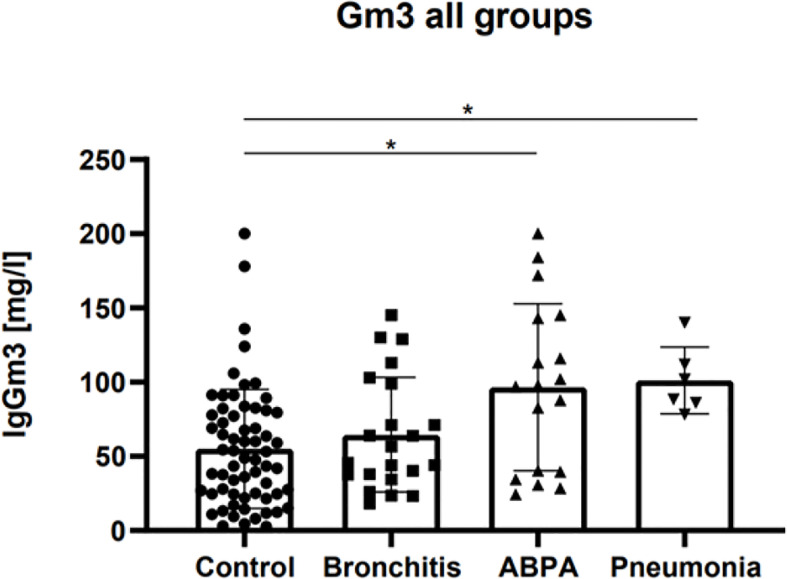
Gm3 (mg/L) for control group, *Af* bronchitis group, ABPA group and *Af* pneumonia group before treatment. Adjusted p-values: control vs. bronchitis: ns, control vs. ABPA: p <0.05, control vs. pneumonia: p <0.05. * represents the level of significance (p ≤ 0.05).

Median Gm3 for the control group was 48.9 mg/L ± 40.15 (2.7 - >200 mg/L), for the bronchitis group 51.2 mg/L ± 38.6 (18.2 - 145 mg/L), for the ABPA group 97.5 mg/L ± 56,34 (24.2 - >200 mg/L), and for the pneumonia group 95.3 mg/L ± 22.5 (78.2 - 140 mg/L, p < 0.01, (see **Figure 1**).

A statistically significant difference was found between median Gm3 of control vs. ABPA group (p < 0.05), and between median Gm3 of control vs *Af* pneumonia group (p < 0.05). No significant difference was found between median Gm3 of control vs. bronchitis group (see **Figure 1**).

In the *Af* bronchitis and ABPA group, several patients did not meet the manufacturer’s cut-off value of >39 mg/L: in the bronchitis group, 31.8% (n=7) patients had Gm3 levels below the cutoff, and in the ABPA group, 22.2% (n=4) patients had Gm3 levels below the cutoff. In contrast, in the control group, a majority of 58.7% (n=37) patients had Gm3 levels above the cutoff. In the *Af* pneumonia group, all patients had Gm3 levels above the manufacturer`s cut-off of >39 mg/L. (see **Figure 1**).

### Follow-Up Gm3

**Figure 2:**
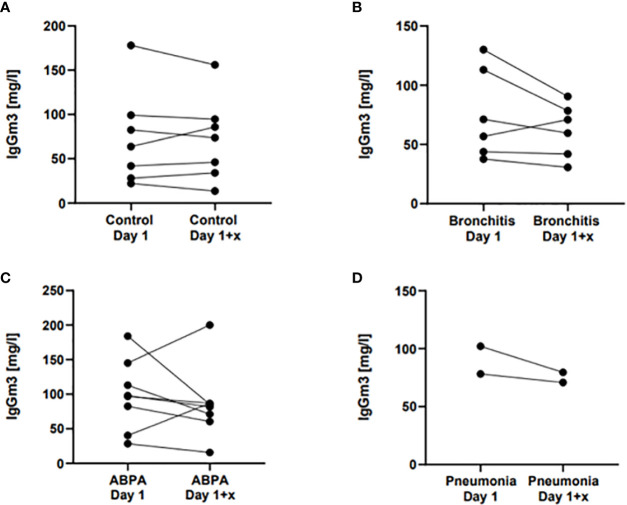
Gm3 follow-up measurements for **(A)** control group, **(B)** *Af* bronchitis group, **(C)** ABPA group and **(D)** *Af* pneumonia group, for each group at time of diagnosis (Day 1) and after treatment (Day 1 +x), for control group at any two consecutive times. Adjusted p-values: no statistical significance.

Twenty-one patients could be enrolled for a follow-up examination. In the *Af* disease groups, it was assessed between 30 and 180 days after diagnosis. For the control group, mean Gm3 was assessed at two arbitrary points in time. The mean difference of the control group was 1.6 mg/L. For the bronchitis group, the mean Gm3 difference after initiation of therapy was 13.4 mg/L, and for the ABPA group, it was 12.6 mg/L (no statistical significance). In both the bronchitis and ABPA group, single patients showed a rise in Gm3 in the follow up (see **Figure 2**).

### Cut-Off Determination

**Figure 3:**
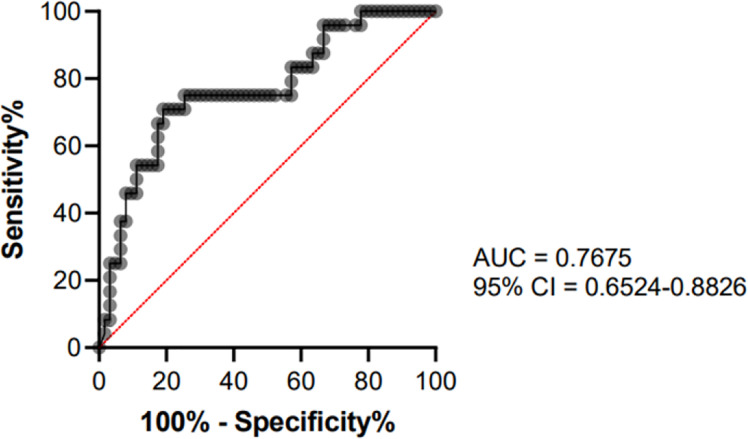
ROC curve analysis showing the performance of Gm3 for distinguishing control group from ABPA/*Af* pneumonia group.

**Figure 4:**
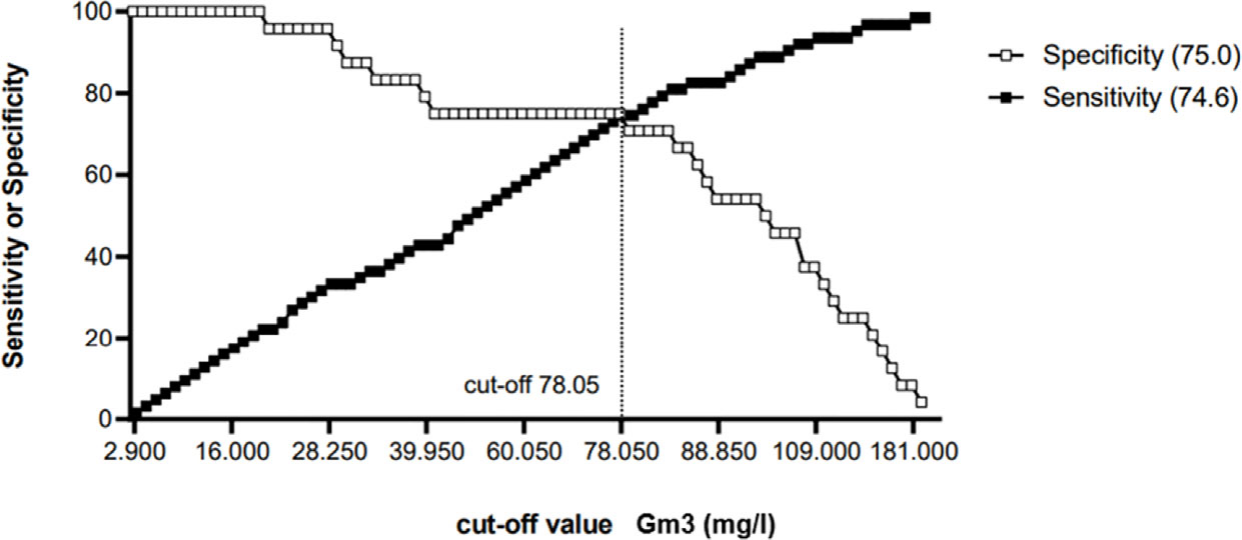
Cut-off-determination: Gm3 = 78.05 mg/L with a specificity (white square line) of 75% and a sensitivity (black square line) of 74.6%.

To determinate a cut-off value, only CF control subjects (n = 63) and CF patients with ABPA or pneumonia (n = 24) were included. ROC curve analysis showed an area under the curve of 0.7675 (95% Confidence Interval = 0.6524–0.88826, see **Figure 3**) A cut-off value of 78.05 mg/L for Gm3 was calculated to discriminate controls and patients with ABPA/pneumonia with a specificity (white square line) of 75% and a sensitivity (black square line) of 74.6% (see **Figure 4**).

## Discussion

In this study, we evaluated the utility of *Af* specific Gm3 for the diagnosis of *Af* associated diseases in CF. Mean Gm3 levels revealed a significant difference between control group vs. ABPA and vs. pneumonia group, but not vs. bronchitis group. *Af* specific IgG may therefore be a useful biomarker for acute ABPA and *Af* pneumonia in cystic fibrosis, but not for *Af* bronchitis in cystic fibrosis.

However, we also found a wide distribution of Gm3 in the control, bronchitis and ABPA group. Thus, for the diagnosis of ABPA and *Af* pneumonia in CF, Gm3 should only be interpreted alongside other biomarkers.

Interestingly, a significant proportion of the Gm3 of our control group exceeded the manufacturer`s cut-off-level of 39 mg/L. Due to the high exposure of the CF lung to respiratory pathogens, the immune response in CF—here in the form of Gm3—may fundamentally differ from that of healthy individuals. Specific cut-offs may therefore be required in CF to describe pathogen-mediated conditions. Positive and even high titers of Gm3 do not necessarily represent *Af*-associated disease. On the other hand, the pathogenic impact and the need for treatment of chronic colonization with *Af* in CF is still under discussion. In our cohort, 33% of the control group patients had *Af* positive sputum (see **Table 1**). There could be cases of bronchitis in the control group that did not meet the definition of bronchitis. Another reason for high Gm3 levels in the control group may be cross reactivity to other fungi colonizing the respiratory tract in CF, as m3 is the crude *Af* antigen and shows cross reactivities to other opportunistic fungi (Fukutomi et al., 2016). In our cohort 85.3% were co-colonized with *Candida* spp. and 11% with other fungi (see **Table 1**). If this problem could be overcome by using recombinant antigens, is unclear, because so far, no investigation has been performed under this aspect. The studies conducted with recombinant antigens to date show vastly different results (Nikolaizik et al., 1995; Kurup, 2005; Fricker-Hidalgo et al., 2010; Alghamdi et al., 2019). Finally, in some patients in the cohort of acute *Af* diseases, Gm3 exceeds the measurement range of 200 mg/L. Quantification above 200 mg/L could therefore improve sensitivity and specificity.

The non-significant difference in median Gm3 levels between control and bronchitis group could be explained by a predominantly endobronchial host-pathogen interaction in *Af* bronchitis, which may less likely lead to IgG production than in the more invasive forms of aspergillosis. This may also explain why a significant proportion of the bronchitis group had Gm3 levels below the manufacturer`s cut-off. On the other hand, also a small but still significant proportion of the ABPA group had Gm3 levels below the manufacturer`s cut-off, which may be due to a predominant Type 1 hypersensitivity reaction in these cases.

Our results of a Gm3 cut-off-level of 78.05 mg/L with a sensitivity of 75% and a specificity of 74.6% in *Af* pneumonia and ABPA in CF differ substantially from those found in other populations. Agarwal et al. examined *Af* specific Gm3 in asthmatic patients with ABPA and *Af* sensitization without ABPA and found a sensitivity of 88% and specificity of 100% when using a cut-off level of 26.9 mg/L ImmunoCAP Gm3 (Agarwal et al., 2017). In chronic pulmonary aspergillosis in non-CF-patients, Sehgal et al. found a sensitivity and specificity of 95.6% and 100% using a Gm3 cut-off of 27.3 mg/L (Sehgal et al., 2018). For simple aspergilloma in pulmonary tuberculosis patients, the same working group found a sensitivity of 63.5% and a specificity of 98.3% using a cut-off of 27.3 mg/L. However, the Gm3 cut-off-level is still under discussion, as a recent multicentric study found a median Gm3 of 21.3 mg/L and a 90% quantile for Gm3 of 78 mg/L in a population of 121 healthy donors (Raulf et al., 2019). The working group suggested that IgG values higher than at least 90% of the healthy donor group indicate elevated levels, which is more consistent with our findings of a cut-off-level of 78.05 mg/L.

Our follow-up data, where some patients showed a rise in Gm3 at a second point of time after diagnosis are consistent with the data of Agarwal et al. in asthmatic patients with ABPA, who observed an increase of Gm3 in 23.1% of the patients and a decrease in 23.1% (Agarwal et al., 2017). However, immunoglobulins are often maintained long-time following antigen contact, and, so far, no longitudinal data exist for titer progression of Gm3. Considering the small number of cases in our cohort, no statement can be made if and after what time Gm3 is suitable as a follow-up marker. Due to the low prevalence of *Af* diseases in CF, the suitability of Gm3 as a screening and outcome parameter should be investigated in a multicenter study.

## Conclusion

*Af-*specific IgG may be a useful biomarker for acute ABPA and *Af *pneumonia, but not for *Af* bronchitis in CF. However, due to the large interindividual variability of Gm3, it should only be interpreted alongside other biomarkers. Therefore, due to its broad availability, it could be suitable as a biomarker for ABPA and *Af* pneumonia in CF, if the results can be supported by a larger multicenter cohort. Standardized, longitudinal studies and a better knowledge of underlying immunological mechanisms may improve its usability.

## Data Availability Statement

The raw data supporting the conclusions of this article will be made available by the authors, without undue reservation.

## Ethics Statement

The studies involving human participants were reviewed and approved by Ethikkommission Charité Universitätsmedizin Berlin. Written informed consent to participate in this study was provided by the participants’ legal guardian/next of kin.

## Author Contributions

PE, CG, and CS contributed to the conception and design of the study. PE and CG organized the database. CG performed the statistical analysis. PE, CG, and CS wrote the manuscript. All authors contributed to the article and approved the submitted version.

## Funding

This work was supported by the German Federal Ministry of Education and Science (BMBF)—Project InfectControl 2020 (“ART4Fun”; 03ZZ0813E).

## Conflict of Interest

The authors declare that the research was conducted in the absence of any commercial or financial relationships that could be construed as a potential conflict of interest.
